# Gas6/Axl in arginine-starvation therapy

**DOI:** 10.18632/oncoscience.218

**Published:** 2015-08-25

**Authors:** Wen-Bin Tsai, Yan Long, Macus Tien Kuo

**Affiliations:** Department of Translational Molecular Pathology, The University of Texas MD Anderson Cancer Center, Houston, TX, USA

**Keywords:** Gas6, Axl, arginine-starvation, ADI-PEG20, melanoma

Exploiting abnormal amino acid metabolism for cancer therapy dated back to more than four decades ago by the discovery that L-asparaginase was effective in treating childhood acute lymphocytic leukemia (ALL). ALL lacks asparagine (Asn) because the key enzyme for the biosynthesis of Asn from aspartic acid, asparagine synthetase, is silenced, therefore, ALL requires Asn from the circulation for growth. Asparaginase (trade names Elspa or Concapar) catalyzes the hydrolysis of Asn in the circulation, resulting in Asn starvation and cell death. Asn starvation therapy has shown remarkable treatment efficacies [1].

Another amino acid starvation therapy strategy that has been under development is the use of pegylated arginine deiminase (ADI-PEG20) which degrades arginine (Arg) into citrulline and ammonia. Arg is an important metabolite in the urea cycle for tumor cell growth. *De novo* biosynthesis of Arg is regulated by the rate-limiting enzyme, argininosuccinate synthetase 1 (ASS1). Reduced levels of ASS1 expression has been found in large subpopulations of solid tumors, including malignant melanoma, hepatocellular carcinoma, prostate cancers, lung cancers and breast cancers. These ASS1^low^ tumors require extracellular Arg to support their highly proliferative activities. ADI-PEG20 treatment causes Arg starvation resulting in tumor cell death of autophagy/apoptosis. ADI-PEG20 has been in clinical investigations for treating ASS1^low^ tumors with promising results [2].

One major problem associated with ADI-PEG20 treatment is drug resistance due mostly to induction of ASS1 expression. Previous studies showed that ASS1 silencing is mediated by an epigenetic mechanism due to DNA methylation at the promoter region of *ASS1* [3]. Tsai et al [4] demonstrated that ASS1 silencing in melanoma cells is transcriptional regulated by repressor HIF-1α which binds to the E-box of *ASS1* promoter. Arg deprivation induces c-Myc expression which de-represses HIF-1α and induces ASS1 expression.

The recent article by Tsai et al [5] presented a comprehensive signaling mechanism of how ASS1^low^ cells response to Arg starvation stress leading to ASS1 activation and ADI-PEG20 resistance. These investigators reported that within minutes after Arg depletion of ASS1^low^ cells, a reactive oxygen species-related mechanism triggers Gas6 externalization to interact with its membrane-bound receptor Axl, which then activates the downstream signal Ras/PI3K/Akt/GSK-3β growth signal, leading to accumulation of c-Myc by protein stabilization. c-Myc can also transcriptionally upregulate itself and Axl, thereby amplifying the Arg stress response signal. Upregulation of ASS1 expression by c-Myc enhances Arg production and relieves Arg-starvation stress pressure; elevated ASS1 can also feedback-downregulate c-Myc and Axl, providing a self-guarding control mechanism for Arg biosynthesis. These results demonstrated that multiple inter-regulatory pathways involving Axl, c-Myc, ASS1 regulate mammalian Arg homeostasis system and sensitivity to Arg starvation therapy using ADI-PEG20. These authors also demonstrated that expression of ASS1, c-Myc, Gas6 and Axl were elevated in primary cultures derived from two melanoma patients failed ADI-PEG20 treatments as correspondingly compared with those derived from the same patients prior to ADI-PEG20 treatment, demonstrating the clinical relevance of the observations.

The mechanism that ASS1^low^ cancer cells use Gas6/Axl growth signal in response to Arg starvation differs from the conserved general amino acid control pathway which involves rapid repressing protein synthesis due to phosphorylation of translation initiation factor 2 (eIF2) [6]. Recent studies have demonstrated that high levels of Gas6/Axl are associated with tumor aggressiveness. Moreover, elevated expression of Gas6/Axl can also affect the treatment efficacies with many cancer chemotherapeutics. Many small molecules targeting Gas6/Axl have been under development [7]. Moreover, Gas6/Axl may serve as important therapeutic markers for Arg starvation treatment. Because Gas6 secretion is the early event associated with Arg deprivation treatment, Gas6 in the liquid biopsy could become a non-invasive therapeutic marker, although a sensitive detection method may have to develop because basal level Gas6 levels in human serum is very low. Thus, the work by Tsai et al [5] provides an important rationale for further research on improving the treatment of Arg-auxotrophic tumors.
